# A Systematic Review of Diagnostic Accuracy and Clinical Applications of Wearable Movement Sensors for Knee Joint Rehabilitation

**DOI:** 10.3390/s21248221

**Published:** 2021-12-09

**Authors:** Robert Prill, Marina Walter, Aleksandra Królikowska, Roland Becker

**Affiliations:** 1 Center of Orthopaedics and Traumatology, Brandenburg Medical School, University Hospital Brandenburg/Havel, 14770 Brandenburg an der Havel, Germany; roland.becker@mhb-fontane.de; 2 Hasso-Plattner-Institut, University of Potsdam, 14469 Potsdam, Germany; mail@marinawalter.de; 3 Ergonomics and Biomedical Monitoring Laboratory, Department of Physiotherapy, Faculty of Health Sciences, Wroclaw Medical University, 50-367 Wrocław, Poland; aleksandra.krolikowska@umw.edu.pl

**Keywords:** wearable movement sensor, IMU, motion capture, reliability, clinical, orthopedic

## Abstract

In clinical practice, only a few reliable measurement instruments are available for monitoring knee joint rehabilitation. Advances to replace motion capturing with sensor data measurement have been made in the last years. Thus, a systematic review of the literature was performed, focusing on the implementation, diagnostic accuracy, and facilitators and barriers of integrating wearable sensor technology in clinical practices based on a Preferred Reporting Items for Systematic Reviews and Meta-Analyses (PRISMA) statement. For critical appraisal, the COSMIN Risk of Bias tool for reliability and measurement of error was used. PUBMED, Prospero, Cochrane database, and EMBASE were searched for eligible studies. Six studies reporting reliability aspects in using wearable sensor technology at any point after knee surgery in humans were included. All studies reported excellent results with high reliability coefficients, high limits of agreement, or a few detectable errors. They used different or partly inappropriate methods for estimating reliability or missed reporting essential information. Therefore, a moderate risk of bias must be considered. Further quality criterion studies in clinical settings are needed to synthesize the evidence for providing transparent recommendations for the clinical use of wearable movement sensors in knee joint rehabilitation.

## 1. Introduction

Knee joint problems are widespread and may occur throughout a patient’s lifespan. Given the high incidence across the age continuum and the frequent need for surgical repair and long-term rehabilitation, knee injuries present one of the highest clinical and public health injury-related burdens [1,2]. Ligament damage to the knee, including the most frequently injured anterior cruciate ligament (ACL), is more common than any other type of knee injury pathology [3,4]. Additionally, knee osteoarthritis (KOA), with its global prevalence, amounts to almost 23% in individuals aged 40 and over [3], and accounts for nearly four-fifths of OA burden worldwide [5]. The incidence of KOA is 203 per 100,000 person-years in individuals aged 20 and over, and it increases with age to peak at 70–79 years old [6]. Although end-stage KOA can be effectively treated with total knee arthroplasty (TKA), the procedure is related to substantial health costs [7,8].

Patients with knee disorders of different natures require a dedicated follow-up involving physicians, nurses, physical therapists, and other medical staff. Therefore, the healthcare sector is facing challenges regarding the rapidly growing elderly population, rising cost pressure, and limited temporal resources of medical staff. New postoperative protocols are well established and have significantly reduced the time of hospitalization. Cost explosion has induced an increasingly shorter inpatient care of surgical patients, which often induces restrictions in rehabilitation and follow-up quality.

Sensing technology is widely used in orthopedics nowadays. Most commonly it is established in intraoperative care and basic science on human movement [9,10,11,12]. Since wearable technology nowadays possesses the capacity for monitoring and diagnostic functionality, this technology might help solve some of the challenges the healthcare sector faces. Current research has indicated that wearable sensing technology can benefit patients’ care. This device helps physiotherapists and orthopedic surgeons detect movement pattern problems, such as asymmetrical limb loading after anterior cruciate ligament reconstruction (ACLR) [13,14] and quantification of varus thrust in patients with KOA [15]. For patients with total knee replacement, some parameters were used to describe the progress of certain selected parameters relevant for rehabilitation, even if not evaluated for this setting. General gait analysis [16], stance and swing phase development [17], range of motion [18], and knee instability before [19] and after [20] were evaluated before and after total knee arthroplasty (TKA).

So far, no wearable sensing technology system has been successfully incorporated into everyday clinical practice or in a hospital or rehabilitative setting. The feasibility of clinical implementation and the possibility of reimbursement by health insurance companies largely depend on usability, cost-effectiveness, availability, and, most important, diagnostic accuracy. To account for this current gap in knowledge, reviews that focus on these aspects would be helpful.

To date, reviews that have tackled the topic of sensor technology in the medical field have investigated the issue from a broader view. A review from 2012 by Patel et al. focused on wearable sensors and systems with applications in rehabilitation [21]. This review provided an overview of different sensing technologies, such as built-in smartphone sensors, ambient home sensory sensors, fabric electrodes, and various types of wearable devices, to measure blood glucose levels, respiratory rate, ECG, etc. Additionally, potential use cases of telemonitoring in the aging population were discussed. Sensing technology and biomedical markers are commonly used nowadays in various fields of medicine, such as stroke rehabilitation [22] or ankle joint power [23], and rehabilitation issues, such as hand-finger orientation, have already been considered [24].

In 2018, Porciuncula et al. provided what they called a “focused discussion” about current sensor technologies and their clinical applications [25]. They did not provide a comprehensive systematic review but provided an overview of clinical applications used in patients with neurological and musculoskeletal diagnoses, which could potentially benefit from wearable sensors during their rehabilitation. They included different sorts of sensors, such as phone-based sensors or those included in shoes or wristbands for activity recognition, identification of pathologic motor features, falls management, and other clinical applications. The most recent scoping review from 2019 provided by Small assessed the current methodology and clinical application of accelerometers and inertial measurement units (IMUs) to evaluate a patient’s activity and functional recovery after knee arthroplasty [26].

The reviews mentioned above provide a broad scope of the topic. However, apart from the review by Small et al. (2019), the issues of patients with knee pathologies have only been covered to a limited extent. Therefore, the current review focuses on diagnostic accuracy and the different approaches of wearable sensing technology used for monitoring knee and lower limb motion in clinical practice.

**Highlights:**

- Promising IMU quality criterion data exist for describing knee joint status
- No wearable sensing technology assessing knee joint rehabilitation issues has been incorporated successfully into clinical practice
- No consensus about added value from IMUs and quality criterion parameter statistics to be reported
- IMUs are currently used to raise the efficiency of established tests but have high potentials for new parameters with higher validity for function

## 2. Materials and Methods

A systematic review was conducted using Preferred Items for Systematic Review and Meta-Analysis (PRISMA) and accordance with recently published author guidelines for Systematic Reviews and Meta-Analyses [27]. The protocol was preregistered at the open science framework: 10.17605/OSF.IO/DQEAX. To be included in this review, papers must report on the use of at least one IMU for assessing knee joint kinematics, knee stability, or gait analysis. Optimally, studies include validation against a gold standard. Included studies were conducted either in a hospital, ambulatory, or gait laboratory setting. The study population underwent either TKA or ACLR as some of the most commonly performed knee surgeries. Wearable sensing technology has become smaller, more efficient, less obtrusive, and increasingly affordable due to advanced technology. This also leads to an increased number of scientific studies in the field in the last months and years. Nevertheless, to capture all potentially relevant research for this very specific systematic research, PUBMED, Prospero, Cochrane database, and EMBASE were screened for papers from 1980 to 13 March 2021. For identification of relevant studies in the English language, a literature search with the keywords “knee” AND “sensors” OR “IMU” OR “inertial measurement unit” in those electronic databases was conducted.

Due to various methodologies among different journals, a comprehensible guideline for inclusion or exclusion criteria was required, as provided in Table 1. Review articles were excluded but examined for potentially relevant research articles. Exclusion criteria included the use of intraoperative sensor technology to enhance surgical outcomes, app-based intervention, and telerehabilitation studies that did not use wearable sensor technology.

Two independent reviewers screened the manuscript titles and abstracts. Exclusion and inclusion criteria, as presented in Table 1, were discussed among reviewers before the title and abstract screening. After searching and title screening the online database resources, duplicates were removed. For the manuscripts that both reviewers included, a full-text search was performed to decide upon inclusion for the review. Exclusion and inclusion criteria were discussed among reviewers before the title and abstract screening. The full-text screening was performed accordingly.

Following the relevant items of the STARD for reliability checklist, data from the included papers were summarized in a data extraction spreadsheet independently by both reviewers. Disagreements were solved via discussion. Data extraction was grouped by patients’ demographics, type of sensing technology, outcome variables, and diagnostic accuracy criteria. An overview of the different testing protocols was included. A COSMIN Risk of Bias tool was used to examine the quality in a systematic and transparent manner [28]. No ethical approval was required since only existing peer-reviewed literature sources were accepted for evaluation. No data registration plan was needed.

## 3. Results

**Figure 1:**
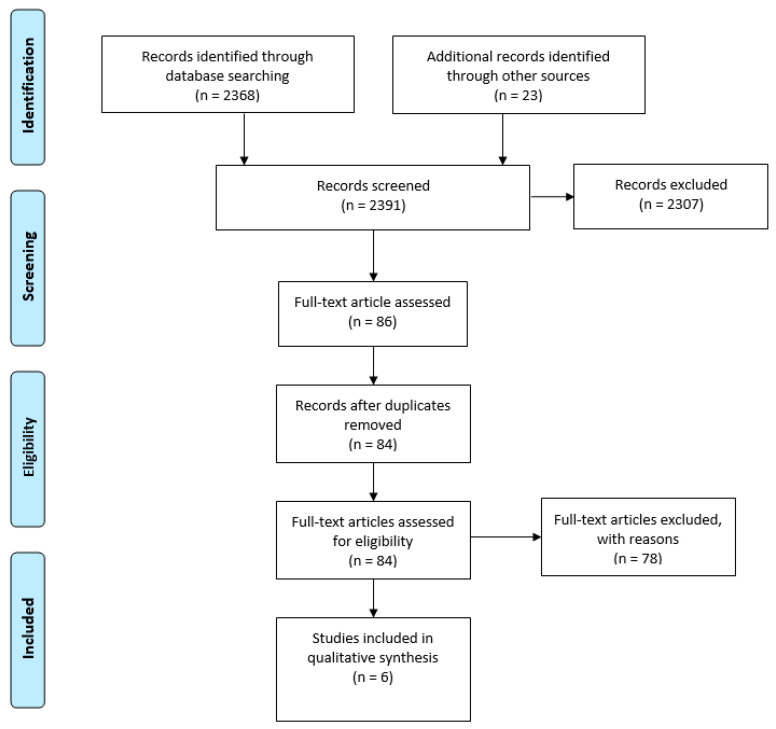
PRISMA flow diagram detailing the results of the literature search and review.

The initial database research with the previously defined search string yielded 2368 results. After the title and abstract search, 84 manuscripts remained and underwent full-text assessment, of which 78 were excluded according to the criteria specified in Table 1. Therefore, six manuscripts remained for inclusion in the qualitative synthesis. Figure 1 shows a PRISMA flow diagram detailing the results of the literature search and review.

Promising study protocols that assess the practical clinical usability of sensing technology have been registered in the last two years. Still, since no results have been published yet, they were excluded from this review. Comparability of studies was limited since various methodological approaches existed. Due to a lack of standardization and an abundance of proprietary solutions, the studies differed regarding sensing technology, dedicated analysis software, sensor placement, testing protocols, and measured outcome variables. Three studies investigated patients who received TKA surgery, and the other three focused on patients after ACLR. The most widely used reference system was the optoelectronic motion capturing system, often not reported in detail, and sometimes complemented with additional force plates.

Different outcome variables were used for patient evaluation after TKA. Temporospatial parameters of gait were measured (cycle time, stance time, and swing time) by De Vroey et al. [29] and knee flexion angles by Roberts et al. [20]. For leg swings, joint instability acceleration-based parameters were measured by Huang et al. [30]. Outcome measures for the ACLR population included gait analysis in one study [14] and knee loading asymmetries with a single limb loading (SLL) task in two other studies [13,31]. Table 2 presents the baseline characteristics of the included studies.

De Vroey et al. [29] used wearable sensing technology to analyze the temporal parameters of gait in a TKA population. The objective was to investigate the agreement between an IMU and a camera-based motion capturing system. Sixteen patients included one year after TKA were asked to perform three gait trials with a self-selected speed along a six-meter walkway. The sensors were placed at the anteromedial facet of the tibia at the left and right lower leg, approximately 5 cm below the knee joint line. Inertial measurement sensor data and optoelectronic camera motion data were collected simultaneously during the gait trials. Custom-made software was used to identify gait events from the gyroscope data. From these data, cycle time, stance time, and swing time were derived. The kinematic data from the camera system were analyzed based on a coordinate-based algorithm. Both sets of temporal variables were compared by calculating intra-class correlation coefficients (ICCs), mean errors, and root mean squared errors. De Vroey et al. found very good to excellent ICC values (0.826–0.972) between the sensor-based and optoelectric motion-based method. The root mean squared errors between both methods ranged from 0.036 to 0.055. Overall, all observed variables showed high levels of agreement. The findings of De Vroey et al. indicated that IMUs can be used in clinical settings to assess temporal gait parameters in the knee arthroplasty population. However, no studies have been published so far proving the usage of the sensors in daily clinical practice.

In a study on monitoring knee flexion angles for rehabilitation purposes in a total knee replacement population, Huang et al. used wearable sensing technology. They compared the measured range of motion between inertial measurement sensors and the Cybex^®^ isokinetic dynamometer (Cybex NORM; Lumex, Inc., Ronkonkoma, NY, USA). The sensor comprised an ATMEGA328 microcontroller, a MPU6050 triaxial accelerometer and gyroscope module, an Arduino Bluetooth module, a lithium battery (9 V, 650 mAh), and a smartphone. The smartphone was used to receive signals transmitted by the Bluetooth module from the accelerometer and gyroscope. The two sensor devices were worn on the thigh and ankle. Thirty-five subjects were enrolled in the experiments, comprising 16 healthy controls and eight patients post total knee replacement. The testing protocol of Huang et al. comprised three indices used as metrics to measure knee rehabilitation progress: number of swings, maximum knee flexion angle, and duration of practice each time. Each subject wore one sensor device on the right shank, and angular speeds of 25, 60, and 180°/s were used, while the swing phase was driven by the Cybex^®^. The system’s accuracy was calculated based on the difference between the detected angle of the sensors and the ROM of Cybex. Huang et al. found that the correlation coefficients between the two measurements at the three angular speeds mentioned above were 0.975, 0.969, and 0.967, respectively. The results indicated high consistency between the sensor-based system and the Cybex reference standard. Correlation coefficients for the TKA subjects, under the same measurement conditions, were calculated to be 0.993, 0.982, and 0.986, again based on three different angular speeds of 25, 60, and 180°/s. Again, this implies a high correlation between the sensor-based system and Cybex. They also found that the average absolute swing errors for the TKA patients were between 1.65° and 3.27°, resulting in accuracies between 96.16% and 98.09%, depending on angular speeds, while accuracies decreased with higher angular speeds of Cybex. Huang et al. concluded that inertial measurement sensors are comparable with professional equipment and, therefore, can be deployed in a clinical setting [30].

Roberts et al. attached a single IMU at the level of the tibial tubercle in patients after TKA and healthy controls. They measured the linear acceleration of the knee joint during several activities of daily living. A direct tibia-mounted accelerometer was compared with a rubber skin-mounted accelerometer in a cadaveric study to ensure skin-mounted devices accuracy. Bland-Altman analysis of acceleration profiles indicated limits of agreement of −0.600 to 1.252 between the two methods. The healthy controls and the TKA cohort were analyzed for statistically significant differences regarding their general activity level, pain for each activity, and instability for each activity. They developed a testing protocol that included five activities of daily living, which were then evaluated with the IMUs and compared against self-reported instability levels. Controls and patients with TKA were found to be comparable regarding general activity scores. Twenty-four out of 38 patients with TKA reported instability during the exercises, with instability depending significantly on the activity performed (*p* = 0.015). Stepping up and down was the most prone to experiencing instability. Furthermore, this was the only activity in which any patient reported severe instability. None of the parameters concerning pain or instability were clinically relevant. Parameters in the y-plane seem most promising, showing extremes in movement [20].

Pratt et al. used wearable sensing technology following ACLR to detect knee power deficits. Their objective was to determine the diagnostic accuracy of inertial sensor thigh angular velocities to detect asymmetrical knee loading. Pratt et al. used two inertial sensors equipped with triaxial accelerometers, gyroscopes, and magnetometers (manufactured by Opal brand, APDM Inc., Portland, OR, USA). The sensors were placed bilaterally on the mid-lateral thighs. Twenty-one individuals following ACLR performed three trials of SLL tasks on each leg while being recorded with a wearable sensor system. Concurrently, the subjects were monitored using an optoelectronic motion capturing system with additional force plates. Pratt et al. calculated between limb ratios for knee power in ACL-reconstructed and contralateral legs based on motion-capturing data. Furthermore, thigh angular velocity was extracted from the inertial sensors, and their ratio was used to diagnose asymmetrical knee loading with receiver operating characteristic curve (ROC) analysis. Asymmetrical knee loading was defined as knee power deficits exceeding 15%. Thigh angular velocity symmetry ratio was discriminated between asymmetrical and symmetrical knee power with high specificity (100%) and sensitivity (81.2%). The study’s findings underlined the feasibility of thigh angular velocities extracted from inertial sensors for clinical detection of knee power asymmetries in individuals following ACLR, allowing for clinical quantification of dynamic knee loading deficits [13]. Furthermore, the authors aimed to prove that knee loading deficits can be identified more easily and with less clinical expenditure using inertial sensor technology. They tried to deduce information about knee moment/knee power (KMom/KPow) during dynamic tasks based on angular velocity measurements with inertial sensors in a cohort of post-ACLR patients. ICCs exceeded 0.947 (*p* < 0.001) for all variables [31].

Sigward et al. explored knee loading asymmetries in individuals after unilateral ACLR using sensor technology too. The authors analyzed the relationship between shank angular velocity and knee extensor moment during a gait trial using an IMU, while validating against a motion-capturing system with force plates. Sigward et al. used two calibrated and synchronized inertial sensors equipped with tri-axial accelerometers, gyroscopes, and magnetometers manufactured by Mobility Lab software, APDM Inc., Portland, Oregon, USA. The inertial sensors were placed bilaterally on the lateral shanks. If the IMU position coincided with that of the MOCAP tracking marker cluster, the IMUs were fixed firmly on top with adhesive tape. Nineteen individuals were instructed to walk 10 m at a self-selected speed. Three trials for each limb were collected. The symmetry between the limbs was calculated using the ratio of peak knee extensor moments of the surgical knee relative to the non-surgical knee. Three trials were averaged for analysis. Sigward et al. found no differences between the limbs regarding stance (*p* = 0.132) and swing (*p* = 0.840) times. However, the peak knee extensor moment and peak shank angular velocity in the ACL-reconstructed knee markedly exceeded those of the contralateral knee (*p* < 0.001). The authors found a strong positive correlation between knee extensor moment and shank angular velocity. Shank angular velocities measured by wearable IMUs can therefore be used to calculate knee extensor moments, while the in-between limb ratios were identified as indicators of knee extensor moment deficits. These findings make wearable IMUs feasible for detecting gait impairment after ACLR. It was concluded that spatiotemporal gait parameters, such as stance and swing time, in an ACLR population normalize sooner than knee loading deficits. What is more, the study indicates that observation of gait deviations by clinicians may not be sufficient to detect rehabilitation progress in subjects following ACLR. Wearable IMUs can account for this gap in rehabilitation progress detection [14]. The extracted data are presented in Table 3.

The risk of bias assessment using the COSMIN Risk of Bias tool is presented in Table 4, showing, on average, a moderate risk of bias for included studies.

## 4. Discussion

All the analyzed studies used commercially available sensor technology, apart from Huang et al., who developed a proprietary solution based on Arduino technology [30]. For data analysis, commercial software was complemented with proprietary solutions, often based on MATLAB, for data analysis purposes. Findings from the studies indicate that IMU usage in rehabilitating the knee surgery population provides reliable data compared to the motion capturing gold standard. Due to various study designs and the resulting methodological differences, a synthesis of evidence is not possible. In Table 5, additionally a summary of sensor issues is provided.

Usability: Experience from De Vroey et al. showed that using IMUs drastically reduces the time needed for data collection and processing. Placement of motion capture (MOCAP) markers took them, on average, 20 min, while sensor placement took 3 min only. Data processing from MOCAP markers took, on average, 40 min per subject and trial. Concurrently, computing gait events from IMU data (with the proposed algorithm) required another 10 min per subject and trial [29]. Huang et al. found that IMUs were well usable since they can be worn without spatiotemporal constraints; they can reduce the frequency of patients needing to return to the hospital for inpatient services and thus save medical expenses. Furthermore, they provide accuracy in monitoring the rehabilitation progress. The sensor devices presented in the study can be easily worn on the thigh and ankle with Velcro and an elastic band, and the number of swings and ROM from each rehabilitation course can be recorded and tracked by users or potentially shared with other medical staff [30]. Roberts et al. underlined the advantages of IMU portability and ease of adaptation to space limitations inherent in clinical follow-up visits after TKA surgery. Furthermore, IMUs are less expensive than other diagnostic tools, such as gait analysis systems and fluoroscopy [20]. According to Pratt et al., IMU utilization should be limited. Although they are less expensive than gold-standard motion capturing systems, they still require a computer and expertise to operate and analyze the data accordingly, leaving the need to develop clinician-friendly technology, especially for placement and calibration [13].

Resolving shortcomings in current rehabilitation practice with IMUs: IMUs offer the potential to extend the existing range of rehabilitation measurements. Knee joint instability after TKA is one of the leading causes of further surgical intervention. Quantification of knee joint instability still lacks objectively quantifiable parameters and is evaluated instead through patient history and physical examination [32]. Roberts et al. identified activities of daily life that can help quantify self-perceived instability in the TKR population using a single tibial-worn IMU and supported Khan et al. [33]. Furthermore, according to a systematic review by Barber-Westin et al., in patients after ACLR, the timing of return to unrestricted sports activities still lacks objective assessment [34]. General recommendations are based on the quantification of muscle strength, stability, neuromuscular control, and general function. Furthermore, there is evidence that, in individuals following ACLR, unilateral deficits may be masked during double-limb performance activities and therefore be overseen in conventional clinical assessments. Isolation of the involved limb with unilateral tasks, such as hopping, should be used to identify deficits in performance [35,36]. Unilateral limb monitoring is hard to accomplish in a clinical context without using MOCAP technology. A possible solution is presented with the IMU-assisted detection of knee loading impairment proposed by Sigward et al. The surgical limb can be separately monitored and, therefore, may offer a new criterion for returning to sporting activities in the ACLR population.

Influencing factors and confounders: Joint angle calculation based on inertial measurement data for human motion analysis remains challenging. In IMU-based human motion analysis, the common problem is that the IMU’s local coordinate axes are not aligned with any physiologically meaningful axis. Within the scope of this review, the decision about the optimal sensor set and sensor position remains unclear [37]. Data from the review showed that sensor placement between the studies varied significantly. Some gave detailed instructions for placement regarding specific anatomical landmarks, while others just vaguely mentioned the body part to which the sensor was attached to. Sometimes additional pictures clarify the sensor placement, but reproducibility is not necessarily provided. Previous studies have shown that the placement of sensors is critical for detecting temporal gait events [37,38]. DeVroey et al. mounted the sensor on the anteromedial surface of the tibia to reduce the chance of soft-tissue artifacts. Compared to other studies, where sensors were mounted to the foot or waist, three authors reported it beneficial for gait event detection when the sensor is placed on the shank since gyroscopic data from shank-worn IMUs show a very distinctive pattern. However, single IMUs attached to the pelvis were shown to miss gait events [29]. Pratt et al. placed IMU sensors on top of MOCAP marker clusters, which provided some standardization concerning their placement. They stated that if their findings are translated to the clinical setting, there will be a need to develop a placement protocol to reproduce sensor placement without these marker cluster plates [13]. Suggestions regarding sensor placement have been made before. Rueterbories et al. published a meta-analysis of sensors and sensor combinations capable of analyzing gait in ambulatory settings and showed a comprehensive overview of sensor devices at different body parts [37]. Furthermore, Storm et al. proposed methods that avoid assuming specific orientations in which the sensors are mounted regarding body segments [38]. To achieve comparability of results, in future research, the standardization of sensor placement should be considered carefully.

Testing protocols contained in this review included different activities, such as gait, ROM measurements, SLL tasks, alongside the performance of various activities of daily life, and therefore impaired comparability as well. Roberts et al. found their testing protocol best suited to detecting significant differences between patients and controls in the sagittal plane since most movement parameters of their tested activities projected to this plane [20]. Sigward et al., who detected impaired knee loading in the ACLR population, used between-limb ratios for their assessment—a widely used method for comparing gait mechanics after surgery. They stated that this is feasible assuming that the non-surgical limb demonstrates normal gait mechanics, which may not be accurate. Nevertheless, this provides the best available frame of reference. Furthermore, Sigward et al. noted that gait mechanics are related to walking velocity and likely influenced by other factors, such as shoe wear or walking surface, which might provide further potential for standardization of testing protocols [14]. Testing protocols, population characteristics, and intervention times differed, leading to a lack of comparability of results, although all the studies induced higher accuracy of sensing devices than the standard measurement methods.

Accuracy issues were verified by Huang et al., especially the accuracy of the sensor devices regarding Cybex when detecting lower limb flexion, and they identified potential reasons for inaccuracies during measurements. Sensor data reception issues arose due to sensors not being worn tightly enough to the leg and, therefore, slide during swings. Another reason is the possible inadequacies of the sampling rate. Their sensor device transmitted their measurements with a frequency of 100 Hz to the connected smartphone, which might induce missed capturing of swing angle and overhead of the smartphone memory due to the trade-off between sampling rate and overhead of the smartphone memory. As a second reason, they discussed vibration from the participant’s leg in cases where they tried to resist their leg being passively swung by the Cybex device. This can induce errors in sensor devices [30]. De Vroey et al., who assessed the temporal gait parameters in the TKA population, traced back measurement deviations to the algorithm used to analyze IMU data. The algorithm showed some variability in detecting gait events compared to actual kinetic detection, likely a consequence of flexion and extension of the metatarsal-phalangeal joints [29]. Nevertheless, these errors in timing estimations were small enough not to be of clinical relevance. Adding to the choice of sensing equipment and placement, different algorithms exist to extract gait events from kinematic IMU data. While most algorithms show good accuracy in normal gait, care has to be taken in the gait-impaired population, where the selection of the appropriate algorithm makes a difference [37]. Bruening et al. compared different algorithms for detecting gait events from kinematic data. They suggested that algorithm choice depended on whether the foot’s motion in terminal swing was more horizontal or vertical for foot strike events. They concluded that algorithms match actual gait events best when selected according to visually distinct gait patterns [39]. Their findings can be applied to routine clinical practice since they identified the most appropriate algorithm for each specific gait pattern. Nevertheless, within this review, only a few authors mention their choice of applied algorithm. Huge varieties of IMU gait analysis algorithms and the lack of consensus for their validation make it difficult for researchers to assess the algorithms’ reliability for specific use cases [40].

The following parameters would help to raise acceptance and facilitate IMU implementation in clinical practice: comparable algorithms, bigger sample sizes for powering the conclusions, strong methods for bias reduction, such as standardized marker application, test–retest designs, the inclusion of more testers and different settings in different stages, and, consequently, reporting Intra Class Correlations and Limits of Agreement.

One of the key problems not solved so far is a valid description and detection of the most relevant parameters for measurement to collect from IMUs for describing rehabilitation after knee surgery. Most benefits from the IMU data will be provided for describing the domain “function”. The gold standard for this domain is the use patient-reported outcome measurements (PROMs) and performance based measures so far, but with few correlation in the early rehabilitation [41]. According to Bolink (2015), PROMs and performance-based outcome measures are, for example, only moderately correlated one year after TKA, probably due to capturing a different dimension of function [42]. As shown in this review, there is widespread usage of sensors for detecting changes in knee joint rehabilitation. Still, many of them were only partly evaluated for quality criteria, probably caused by a lack of consensus on relevant parameters for describing the function of the knee joint and related rehabilitation progress. It seems obvious that, in clinical practice, sensors have mostly been used to express existing tests and parameters in an easier or faster way. Bigger advantage from implementing sensors will probably be given when developing new parameters. It might be of value to expand the scope of potentially relevant parameters first, highlighting the value of wearable sensing technology unlike standard performance-based measures in the past. New parameters such as “whole day knee joint angle movement”, “all day stairs used”, or “average limb loading while walking” are currently not reported and might provide higher correlation to patient-reported function and, therefore, broader acceptance among stakeholders, thereby inducing more explicit quality criteria studies in the field. This might lead to consensus discussions and the establishment of core domain sets for this field in addition to existing outcome sets for total knee arthroplasty [43].

## 5. Limitations

Although the studies included in this review showed a wide variety in their approaches, test protocols, and study population, some valuable information can be derived from them. Multiple studies mentioned limited applicability due to the relatively small sample size [13,31]. Furthermore, Pratt et al. emphasized restricted applicability of results since their established testing paradigm can only be applied to individuals four to six months post-surgery who are progressing back to running. Other phases of rehabilitation remain unexplored. Translation of Pratt’s findings cannot be assumed to be widely translatable to different tasks than SLL, which leaves the need to assess other dynamic tasks, such as running. Roberts et al. drew limitations regarding the assessment of tibia and femur motion, respectively, and proposed using two IMUs for better characterization of the relative motion between the two bones to assess the movement of the knee implant parts. They stated that these dynamics might differ in patients with bilateral TKA, unlike those with unilateral one [20]. Meta-analysis was inappropriate because studies were not similar enough from a methodological and clinical viewpoint. No grading of evidence for a specific outcome was possible because of the different topics covered in the included studies. From our point of view, many studies close to the topic had to be excluded, caused by strict inclusion criteria. Reviews on similar topic especially on new potential parameters should be performed.

## 6. Conclusions

The present review shows that IMUs offer sufficient accuracy to replace, combine, and extend the existing range of rehabilitation devices. IMUs can subsume different measures for rehabilitation by assessing outcomes that would typically be measured individually, such as ROM, gait analysis, and detection of asymmetric knee loading, while adding new rehabilitation hallmarks, such as quantification of instability. IMUs can replace time-consuming equipment such as motion-capturing systems and force platforms in the knee surgery population. Developing clinician-friendly, standardized applications of IMUs for clinical practice is imperative. However, all the data provided were collected in a laboratory environment. Furthermore, studies regarding sensing technology utilization in clinical practice remain lacking. Since this technology provides evidence to benefit patients and healthcare providers, its translation into clinical rehabilitation practice is imperative. Some interesting work was done to clarify the diagnostic accuracy of wearable movement sensors for knee joint rehabilitation. Still, in the current stage, comparable quality criterion studies are lacking for an evidence summary of potential measurement bias and clear recommendations for using wearable movement technology in quantifying knee injuries in clinical settings. Developing a core measurement set for quality criterion studies on IMUs for medical use might help harmonize research in knee joint rehabilitation. Generally, within the scope of this review, although there are distinct limitations of sensor usage in rehabilitating knee surgery populations, the potential of these devices is obvious.

## Figures and Tables

**Table 1:** Inclusion and exclusion criteria.

Inclusion Criteria	Exclusion Criteria
Studies including patients with knee osteoarthritis, total knee arthroplasty, or anterior cruciate ligament reconstruction	Studies including intraoperative sensors for enhancing surgical outcomes, such as using pressure sensors for total knee replacement
Studies including patients investigated with at least one IMU	Studies that perform postoperative digital interventions or telerehabilitation without using wearable sensing technology
Studies including body-mounted sensors	Cadaveric studies
Some form of quality measurement of the data needs to be provided	Studies including patients with neurological or rheumatic diseases that impaired balance or ability to walk
	Study protocols

**Table 2:** Baseline characteristics.

	TKA (*n*)	TKA % Female	ACLR (*n*)	ACLR % Female	Healthy Controls	Healthy Contr. % FEM.	2–3 Months *p*. Surgery	4–6 Months *p*. Surgery	>6 Months *p*. Surgery	Gait Analysis	SLL	Other	Mocap	Force Plates	Other
De Vroey	16	50							√	√			√		
Huang	8	75			16	50			√			√			√
Pratt a			21	57				√			√		√	√	
Pratt b			21	57				√			√				
Roberts	27	59			18	61			√			√			
Sigward			19	74			√			√			√	√	

ACLR, anterior cruciate ligament reconstruction; *n*, number of individuals in a given sample; TKA, total knee arthroplasty.

**Table 3:** Data extraction, sensor information, and results.

	Sensor Information and Application	Knee-Joint Measurement Method	Results
De Vroey (2018)	Gyroscope data:	Shank worn	ICC = 0.826–0.972
	Three gait trials	IMUs	RMSE = 0.036–0.055
	6 m walk; TKA patients		
Huang (2020)	Three axial accelerometer and gyroscope data: Number of swings,	MPU6050, ATMEGA328	Measurement error = 1.65°–3.27°
	ROM knee flex, duration, TKA patients, and controls	Cybex	
Pratt (2018a)	Shank gyroscope, maker-based motion and force plate data: Sagittal plane peak knee power	Opal APDM, Qualisis AB, AMTI	81%, Specificity 100% for asymmetrical knee loading
	absorption, ACLR patients		
Pratt (2018b)	Shank gyroscope, knee moments, knee power (angular velocity): single limb loading tasks, ACLR patients	OPAL APDM, Qualisis	ICCs (>0.947); r = 0.81 for thigh and r = 0.54 for knee velocity
Roberts (2013)	Tibial tuberositas IMU; joint acceleration, Jerk: Joint stability, 5 activities on one leg and the other, TKA patients and controls	Motion Nod, gyroscope	Differences (*p* > 0.05) in 22 IMU parameters between patients and controls
Sigwards (2016)	Shank angular velocity and knee extensors movement during gait	Opal APDM gyroscope, Qualisis, AMTI	Peak velocity and knee extensor movement correlate with r = 0.75

ICC = intraclass correlation, RMSE = root mean square errors, ROM = range of motion.

**Table 4:** Risk of bias assessment (consensus results).

	Design Requirements	Stability of the Patients	Time Interval	Similarity of Measurement	Administration without Knowledge of Scores	Score Assignment or Determination of Values	Other important Flaws	Statistical Methods	For continuous Data ICC	For Ordinal: Kappa	For Nominal: Kappa for each Category	Final Rating
De Vroey		√	NA	√	√	√	-		√	-	-	A
Huang		√	NA	√	√	√	-		√	-	-	A
Pratt a		√	NA	(√)	√	√	-		NA			DF
Pratt b		√	NA	√	√	√	-		√			A
Roberts		√	NA	(√)	√	√	-		NA			DF
Sigward		√	NA	√	√	√	-		NA			DF

NA = not available or wrong, (√) = correct, but unclear, A = adequate, DF = doubtful.

**Table 5:** Sensor summary.

	Number of Wearable Sensors	Accelerometer	Gyroscope	Magnetometer	Additional Force Platform	Not Reported	100–200 Hz	50–100 Hz	Commercial Software	Proprietary Solution	Not Described	Leg	Hip	Not Described	Commercial Sensor	Proprietary Sensor
De Vroey	2							√		√			√		√	
Huang	2	√	√			√					√	√				√
Pratt a	2	√	√	√	√			√	√	√		√			√	
Pratt b	4	√	√	√		√			√	√				√	√	
Roberts	1	√	√	√				√		√		√			√	
Sigward	2	√	√	√	√		√		√	√		√			√	

## Data Availability

All extracted data are included in the manuscript.
